# Changes in the Histological Structure of Adrenal Glands and Corticosterone Level after Whey Protein or Bee Pollen Supplementation in Running and Non-Running Rats

**DOI:** 10.3390/ijerph20054105

**Published:** 2023-02-24

**Authors:** Karolina Frankowska, Michał Zarobkiewicz, Mirosław A. Sławiński, Ewelina Wawryk-Gawda, Monika Abramiuk, Barbara Jodłowska-Jędrych

**Affiliations:** 1 Chair and Department of Histology, Embryology and Cytophysiology, Student Scientific Association, Medical University of Lublin, 20-080 Lublin, Poland; 2 First Chair and Department of Oncological Gynaecology and Gynaecology, Student Scientific Association, Medical University of Lublin, 20-081 Lublin, Poland; 3 Chair and Department of Clinical Immunology, Medical University of Lublin, 20-093 Lublin, Poland; 4 Chair and Department of Histology, Embryology and Cytophysiology, Medical University of Lublin, 20-080 Lublin, Poland; 5 Department of Paediatric Pulmonology and Rheumatology, Medical University of Lublin, 20-093 Lublin, Poland; 6 First Chair and Department of Oncological Gynecology and Gynecology, Medical University of Lublin, 20-081 Lublin, Poland

**Keywords:** Wistar rats, whey protein, bee pollen, adrenal glands, stress

## Abstract

Due to the many health-promoting properties of bee pollen and whey protein, both products are widely used as dietary supplements. According to these reports on their health-promoting properties, the aim of our study is to assess whether these products can influence the structure and function of the adrenal glands in rats. Thirty male Wistar rats were divided into six equal groups. Among them, there were three groups which included non-running rats and three groups which included running rats. Both of these running (n = 3) and non-running (n = 3) groups included non-supplemented (control groups), bee-pollen-supplemented groups, and whey-protein-supplemented groups. After 8 weeks, the rats were decapitated, their adrenal glands were collected, and paraffin slides were prepared. Then, staining according to the standard H&E and Masson’s trichrome protocols was performed. Fecal and urine samples were collected prior to the end of the study to measure corticosterone levels. In the group of non-running rats, the consumption of bee pollen was noted to be significantly higher when compared to the group of running rats (*p* < 0.05). The thickness of the particular adrenal cortex layers was similar among all of the groups (*p* > 0.05). The statistically significant changes in the microscopic structure of the adrenal glands, especially regarding cell nuclei diameter and structure, as well as the architecture of sinusoids, were observed between the groups. Moreover, urine corticosterone concentrations were found to vary between all of the analyzed groups (*p* < 0.05). These results indicate that both bee pollen and whey protein have limited stress-reducing potential.

## 1. Introduction

Stress is defined as a physiological response of the body to a state of danger. Such modification of homeostasis is achieved as a result of the complex interactions within the elements of the hypothalamus–pituitary–adrenal axis (HPA axis). As a subsequent consequence of axis activation, adrenal glands—the main organs involved in stress-response—secrete stress hormones, finally regulating the organism functioning at the multi-organ level [1,2].

The occurrence of stress reactions is conditioned multifactorial—both by internal and external stimuli. For sure, any differences in physical activity—its abandonment or limitation—are a possible source of stress induction [3,4]. Moreover, physical activity is also significant from the perspective of counteracting subsequent negative stress effects [5]. It is especially important taking into consideration that exposure to stress has many health consequences and may affect among others the cardiovascular system, the nervous system, or the immune state. The current literature indicates a significant role of psychological stress in the pathogenesis of asthma, Alzheimer’s disease, and cancer development, although due to the complex relevant determinants of these diseases, it is difficult to assess the exact role of stress in their etiology [6,7,8,9]. In addition to triggering the onset of disease, stress modifies the amount and type of food intake, which has been observed both in humans and animal models [10]. The occurrence of some of such changes in food preferences is explained by the influence of corticosterone produced by the adrenal glands [11]. Although the effect of stress on food preferences and the amount and type of consumed food varies, stress can exacerbate the desire to eat palatable foods, rich in fats and sugars, referred to as comfort food [12,13]. Due to the high prevalence of negative stress-related effects in people, substances with beneficial effects on health are being sought to minimize the negative consequences of stress. Hence, it has been proven that different types of food, e.g., coconut oil and mung beans have potential anti-stress values [14,15].

Bee pollen, a product obtained from honeybees, is a very complex compound consisting of approximately two hundred different substances. Although bee pollen composition differs depending on the species from it which originates, all major components, such as proteins, lipids, carbohydrates, or vitamins, and bio-elements are present in bee pollen independently of the origin [16]. When analyzing the percentage share of each group of compounds, it is noticeable that the largest part of it is carbohydrates, the content of which reaches about 30 percent. The average protein content in bee pollen oscillates to and from approximately 20 percent, of which essential amino acids represent a significant proportion. Among the other components of bee pollen are nucleic acids, lipids, and crude fiber [16,17]. In addition, bee pollen is a source of numerous macro- and microelements with a particularly significant content of potassium, iron, phosphorus, magnesium, and zinc [17]. Moreover, its composition is characterized by the presence of practically all vitamins including provitamin A, vitamin E, thiamine, niacin, pantothenic, nicotinic, and folic acid. Among the bioactive substances contained in bee pollen, the presence of phenolic compounds is worthy of note, since flavonoids, which make up a significant proportion of that group of compounds, are responsible for the antioxidant properties of bee pollen [17,18]. Such a rich composition of bee pollen is responsible for a number of the known health benefits of this substance, including hypolipidemic and glucose-ameliorating activities, as well as detoxifying and anti-inflammatory action [17,19]. All of these nutritional properties make bee pollen a valuable functional food able to enrich the diet [19,20].

Whey protein is a substance representing a significant proportion of the proteins contained in cow milk. It is processed to produce preparations such as whey protein concentrate (WPC), whey protein isolate (WPI), or whey protein hydrolyzed (WPH) with varying protein contents [21]. Regardless of the processing route, whey protein is primarily a rich source of β-lactoglobulin and α-lactalbumin. In addition, it is characterized by a content of ingredients such as essential amino acids, branched-chain amino acids, immunoglobulins, and lactoferrin [22,23]. Currently, whey protein is widely used as a supplement among athletes due to its beneficial effects on muscles [23,24]. Its anti-inflammatory, cardioprotective, neuroprotective, and anti-cancer properties also support its role as a functional food [23,24,25].

Although to date, there are no studies on the anti-stress potential of bee pollen, there are reports on the effect on stress of similar products such as propolis and royal jelly, suggesting a potential anti-stress effect in different animals [26,27,28,29,30]. Furthermore, a protein-rich diet is also known for its potential anti-stress properties [31]. In view of these reports, we have investigated whether there is a possibility that bee pollen and whey protein supplementation may influence histological properties, the function of adrenal glands, and thus also the response to stress [32].

## 2. Materials and Methods

### 2.1. Study Protocol

**Figure 1:**
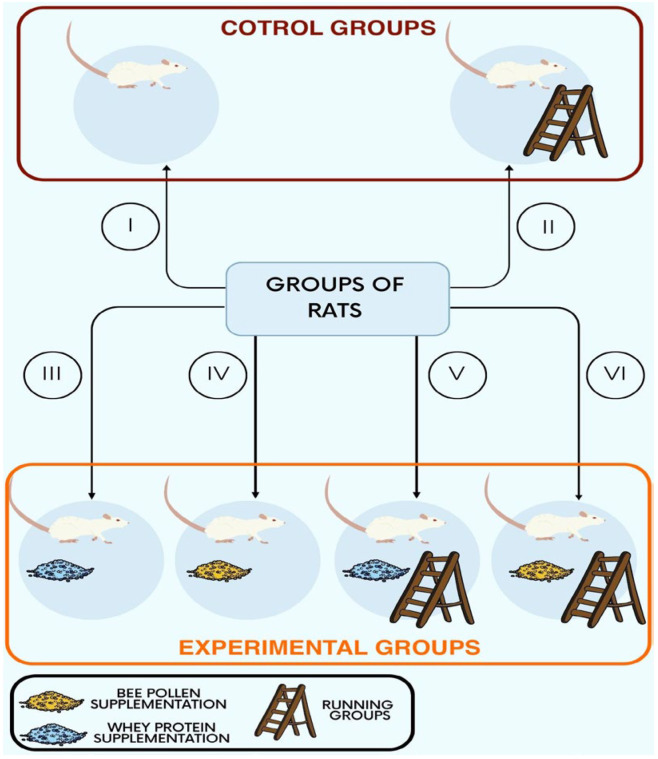
Schematic classification of rats into six groups (n = 5 per group)—two control groups without any supplementation and four experimental ones. The numbers correspond to the group numbers used on the diagram: I—non-running, non-supplemented group (control); II—running, non-supplemented group (control); III—non-running, whey-protein-supplemented group; IV—non- running, bee-pollen-supplemented group; V—running, whey-protein-supplemented group; VI—running, bee-pollen-supplemented group.

Thirty eight-week-old male Wistar rats were divided into six equal groups (five rats per group). Non-supplemented groups (No. I and No. II), also referred to as the control groups, included a non-running group (No. I) and a running group (No. II). The experimental groups (No. III–VI), understood as the supplemented ones, were non-running (No. III–IV) supplemented with whey protein (No. III) or bee pollen (No. IV), as well as running (No. V–VI) supplemented with whey protein (No. V) or bee pollen (No. VI) (Figure 1). During the 8 weeks of the experimental phase, all of the animals received water and rodent food ad libitum; the bee-pollen-supplemented group also received bee pollen and the whey-protein-supplemented group also received enriched whey protein concentrate (Olimp Laboratories Sp. z.o.o., Dębica, Poland). The daily rodent food, bee pollen, whey protein, and water consumption were measured each day. During the experimental phase, the rats in the running groups ran five times per week, with the duration of a single run being 5 min, on a treadmill built by us before starting the experiment. The average velocity was 6 km/h and the rats were not assisted by electrical shock. The rats from the non-running groups did not use the treadmill. At the beginning of the experiment, the body mass of the rats was approximately 330 g, while at the end of the experiment, it increased to approximately 400 g, regardless of the group. At the end of the experimental phase, all of the rats were decapitated and their adrenal glands were collected. Immediately after collection, the adrenal gland mass was measured with a digital analytical balance with 0.1 mg readability AS 110.R2 (Radwag, Lublin, Poland). After fixation in formalin, paraffin blocks were prepared.

The study protocol was approved by the Bioethical Committee at the Medical University of Lublin (No. 24/2015).

### 2.2. Supplements

The bee pollen was collected in the vicinity of Lublin, Poland. It contained approximately 31 g of carbohydrates, 23 g of protein, 5 g of lipids, and 0.8 g of various vitamins (A, E, D, B1, B2, B3, B5, B6, B7, and C) per 100 g [16]. The 100 g of enriched whey protein concentrate (further called either whey protein or WPC) contained 77 g of protein, 6 g of carbohydrates, and 7 g of lipids, as reported previously [33].

### 2.3. Histological Staining and Analysis

Five μm-thick slides were prepared and stained according to the standard H&E and Masson’s trichrome protocols. The slides were then analyzed under a light microscope. Olympus BX4 with a digital camera and CellSens software (Version 4.1. CS-EN-V4) were used for image capture. The measurement of vacuolization was performed in Fiji as previously described [34]. The vacuolization rate was calculated as a percentage of the area occupied by vacuoles to the total analyzed area in the particular cortex layer. The measurement of the extent of fibrosis was performed in Fiji, as reported previously [33].

### 2.4. ELISA

A corticosterone ELISA kit (Cayman Chemical, Ann Arbor, MI, USA) was used for the measurement of fecal corticosterone content. Fresh feces samples, collected during the second to last week of the experiment, were frozen at −80 °C immediately after collection. Prior to corticosterone measurement, the samples were dried in a heat cabinet at 30 °C for 2 h as proposed by L. Pihl and J. Hau [35]. Then, the samples were prepared according to the manufacturer’s instructions. Large particles were removed by shifting through a stainless steel mesh. Twenty mg of each sample was suspended in 1ml of methanol. Then, the samples were vortexed for 30 min and centrifuged for 20 min at 2500× *g*. The supernatant was transferred into clean a Eppendorf tube and diluted 1:50 in ELISA Buffer (Cayman Chemical, Ann Arbor, MI, USA). The samples were prepared in such a way were then used in ELISA according to the manufacturer’s protocol. The absorbance was measured with a plate reader Biotek Elx-800 (BioTek, Winooski, VT, USA).

A corticosterone ELISA Kit (R&D Systems, Minneapolis, MN, USA) was used for corticosterone measurement in urine. Each sample was run in duplicate. Fresh urine samples were collected from metabolic cages shortly prior to study termination and immediately frozen at −80 °C. Prior to analysis, the samples were first transferred to −20 °C and then completely thawed. They were centrifuged for 10 min at 18,000× *g*. The supernatant was collected and diluted 100-times with Calibrator Diluent RD5-43 supplied as part of the kit. An ELISA assay was prepared according to manufacturer’s instructions. The microplate was read with Biotek Elx-800. The raw data were analyzed with elisaanalysis.com (ElisaKit) in the case of urine and with a spreadsheet supplied by Cayman Chemicals in the case of feces. Each sample was run in duplicate.

### 2.5. Statistical Analysis

The collected data were statistically analyzed with Statistica 12 (StatSoft, St. Tulsa OK, USA). The distribution of the data was analyzed with the Shapiro–Wilk test. The statistical significance was calculated with the Kruskal–Wallis test, and the level of significance was set at *p* < 0.05.

## 3. Results

### 3.1. Food Intake

The daily whey protein intake was similar in both the running (5.19 g per rat) and non-running (5.18 g per rat) groups, while the consumption of bee pollen varied with 13.45 g per rat in the non-running group and 11.96 g per rat in the running group. The detailed consumption and mass changes have been reported previously [33].

### 3.2. Adrenal Gland Mass

No statistically significant difference in adrenal gland mass was observed (*p* = 0.65). Specifically, no differences were noted between any of the experimental groups or their respective control groups. The two controls also did not differ from one another either.

### 3.3. Vacuoles and Structure

No significant changes in adrenal architecture were noted under the optical microscope after H&E staining at 400× magnification. In addition, no statistically significant changes in the contribution of lipid droplets to the total selected area within the different layers of the cortex were observed but the experimental groups supplemented with bee pollen exhibited a slight decrease in the vacuolization of both zona glomerulosa and zona fasciculata (Table 1).

The initial evaluation revealed possible differences in the diameter of the nuclei which was later confirmed by detailed measurements (Table 2). Next, the sinusoids were assessed. The differences in the sinusoid width between the groups were statistically significant (Table 2). Sinusoid epithelium thickness was decreased in both of the non-running experimental groups in comparison to the non-running control group. On the contrary, there was a tendency to increase the sinusoid epithelium thickness in the supplemented running groups compared to the running control (Table 2). The sinusoid epithelial cell nucleus diameter tended to increase in the bee-pollen-supplemented non-running group compared to the non-running control group. The capsule thickness increased in all four experimental groups in comparison to the control groups (Table 2).

### 3.4. Fibrosis

Neither visual evaluation nor computed analysis revealed significant fibrosis in any of the groups except for the bee-pollen-supplemented running group. In the latter, mild fibrosis was noted in all of the layers of the adrenal cortex. Additionally, the results of the computed analysis indicated a decrease in collagen fibers in the bee-pollen-supplemented non-running group in comparison to the non-running control group (Table 3).

### 3.5. Corticosterone Production

The urine corticosterone concentration significantly differed in all four experimental groups—both of the tested not-running groups (III—whey-protein-supplemented and IV—bee-pollen-supplemented) exhibited values lower than the non-running control, while both of the tested running groups (V—whey-protein-supplemented and VI—bee-pollen-supplemented) scored higher than the running control (Table 4). Moreover, in the whey-protein-supplemented groups, both in the running and non-running groups, lower values of urinary corticosterone level were observed in comparison to the bee-pollen-supplemented groups (Table 4). Furthermore, the non-running control group showed higher urinary corticosterone levels than the running control group (Table 4). No statistically significant changes in feces corticosterone concentration and total daily corticosterone excretion were noted (Table 4).

### 3.6. Pyknotic Nuclei

**Figure 2:**
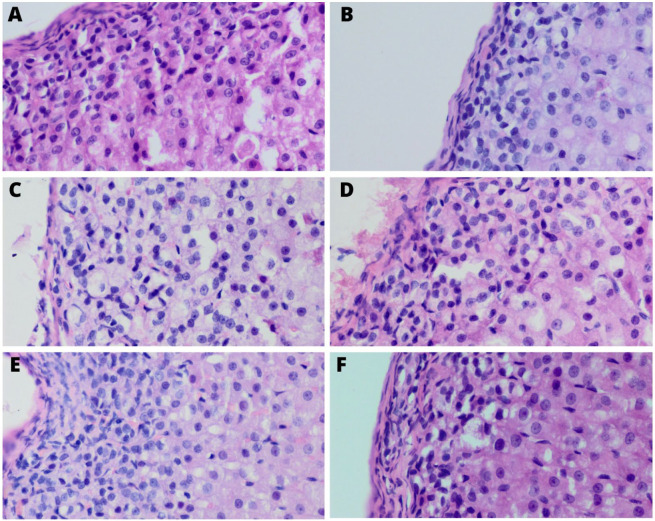
Adrenal glands 400x magnified; non-running control group (**A**); running control group (**B**); non-running whey-protein-supplemented group (**C**); non-running bee-pollen-supplemented group (**D**); running whey-protein-supplemented group (**E**); running bee-pollen-supplemented group (**F**). Visible decrease in pyknotic nuclei in the non-running experimental groups (**C**,**D**) in comparison to the non-running control one (**A**). All of the columns show zona glomerulosa and zona fasciculata. H&E staining.

In all zones of the adrenal gland cortex, statistically significant changes in the percentage of pyknotic nuclei were observed in particular groups. In the non-running experimental groups, supplementation with both bee pollen and whey protein resulted in a lower mean percentage of the pyknotic nuclei in the cells in all of the adrenal cortex zones in comparison to the non-running control group (Table 5), (Figure 2).

## 4. Discussion

To the best of the authors’ knowledge, this is the first study on the effects of bee pollen and whey protein supplementation on adrenal function and structure. The impact of stress on eating behavior is a very complex phenomenon. Depending on the severity of stress and its duration, changes observed in dietary habits are substantially different. The efficient regulation of the whole process especially in the case of chronic stress is provided by the comprehensive action of the hypothalamic–pituitary–adrenal (HPA) axis. Stress affects both the amount and the tendency to eat specific foods as observed both in human and animal models [10,36,37]. While acute stress usually results in reducing food intake, exposure to chronic stressful stimuli leads to an increased desire to consume food. In addition, the regulation of food intake is influenced by the reward center, which in response to palatable food consumption decreases the activity of the HPA axis, resulting in the suppression of the stress response. It may explain why rats in stress are usually more likely to consume sugar-rich comfort products [38,39].

The nutritional trends in rats observed in the current study indicated no change in whey protein intake between the running and non-running groups of rats, whereas the difference in bee pollen intake between these groups was statistically significant. Rats in the non-running group tended to consume more bee pollen compared to the running rats. When analyzing the possible reasons for these observations, it is worth considering the differences in composition in these two groups of palatable food. Bee pollen has a much higher carbohydrate content than whey protein and it has been already reported that rats in stressful situations increase their intake of sugar-rich foods [39].

Consistent with the aforementioned findings, the body’s response to stress is inextricably linked to the functioning of the HPA axis. Especially adrenals, due to their high plasticity, are most significantly modified morphologically and functionally in response to stress [40]. Hence, this is why we decided to evaluate the adrenal glands for morphologic and functional aspects.

In the current study, no changes in the adrenal glands’ weight were observed between the groups. Our findings are consistent with several previous publications evaluating the effects of stress on adrenal mass. Marin et al., (2007) did not observe any changes in adrenal glands’ weight after exposure to stress or chronic restraint [41]. Similarly, it has previously been reported that exposure to chronic variable stress also did not induce changes in adrenal mass [42]. On the other hand, Díaz-Aguila et al., (2018) reported that the combined effect of stress and a high-sucrose diet on the rats caused an increase in the mass of the right adrenal gland. However, this study showed that these two variables separately did not affect adrenal weight which is partly consistent with our results [43]. Nonetheless, there are also reports contrasting with the above conclusions, indicating that stress can increase adrenal weight in rats [44,45,46]. Such an increase in adrenal weight may be due to stimulation of the adrenal glands by ACTH resulting in hypertrophy of the organ [46].

Exposure to stress in rats may lead to changes in the thickness of the adrenal cortex layers—both thinning and thickening are possible [43,47,48]. However, in the present study, microscopic evaluation of adrenal cortex layers in rats in particular groups did not show statistically significant differences.

We found out that the differences in the mean diameter of the cell nuclei in all of the cortex layers and in the medulla between all of the groups were statistically significant. Moreover, we observed a noticeable tendency to increase in the mean diameter of the cell nuclei in the zona glomerulosa and zona fasciculata both in the non-running supplemented groups compared to the non-running control as in the running supplemented groups compared to running control. Increased nucleus diameter may be a sign of increased protein synthesis and may also be related to increased hormonal secretion [49]. The findings previously o0btained from rat models have indicated that changes in nuclei structure are the result of experiencing stress. In a study conducted by Zaki et al., (2018), stress resulted in increased nuclear pyknosis [48] and our results are in line with these statements as we noticed that supplementation with our comfort foods (whey protein and bee pollen) lowered the number of pyknotic nuclei. Similarly, recent experiments have shown the protective effects of whey protein on hepatic cell nuclei [50] and propolis on olfactory bulb [51].

Although there are reports that acute stress induces adrenal fibrosis, in our experiment there is no evidence thereof [52]. Perhaps this is due to the fact that in our experiment, the stress situation was chronic. Nevertheless, we found mild fibrosis of all layers of the adrenal cortex in the group of running rats supplemented with bee pollen. Additionally, computed analysis showed that bee pollen supplementation in non-running groups resulted in a decrease in collagen fibers in comparison to non-running control group. Our results suggest a potential fibrosis-reducing effect exerted by bee pollen. This has been confirmed by previous reports suggesting that another bee product—bee bread—has the ability to reduce liver fibrosis induced by a high-fat diet [53].

Further microscopic analysis also provided observations on the degree of vacuolization of individual layers of the adrenal cortex in each group. The tendency to decrease the level of vacuolization accompanying reduced corticosterone levels according to Koko et al., (2004), can be the result of exposure to short-term stress [52]. The measurement of corticosterone levels in urine and feces is a sensitive marker reflecting the adrenal condition and the level of stress in the body of rats [54]. In the present study, significant differences in corticosterone concentrations were observed only in urine measurements, whereas no differences were found in measurements from stool samples. It has been proven previously that corticosterone concentrations in urine samples reflect the diurnal secretion profile of the hormone [55]. Thus, analysis of changes in urinary corticosterone levels captures a likely picture of stress levels in rats. The differences in the urinary corticosterone concentrations observed in the current study indicate that additional movement was effective in minimizing stress in rats. These results are in line with the reports that restriction of movement in rats caused an increase in blood corticosterone levels [56]. Similarly, our results corroborate those of a previous study in which immobilization and restriction to small space resulted in an increase of urinary corticosterone levels [57]. In addition, Premack et al., (1963), showed that depriving rats of their daily activities leads to an increase in food intake, confirming our previously analyzed model of stress as an inducer of changes in food intake [58].

Moreover, the results of the present study show that, compared to the non-running control group, the supplemented non-running rats had a reduction in urinary corticosterone excretion. This suggests that stress was decreased by both bee pollen and whey protein consumption by rats. The present findings corroborate previous research on the impact of high-protein high-carbohydrate comfort food on the level of stress in animals [31]. Previous studies have shown that royal jelly (another bee product) has the ability to lower plasma corticosterone levels [29,30]. Additionally, an experiment conducted by Teixeira et al., (2017) using royal jelly showed that this product has the ability to reduce corticosterone levels in rats also when they are not under stress [30]. Similarly, an experiment conducted on broilers proved that propolis, another product of bee origin, attenuates the endocrine component of the stress response by lowering corticosterone levels in broilers that were kept in stressful conditions [28]. Thus, the common anti-stress effect of bee products may be based on the activity of one of the proteins contained in them capable of inhibiting cholesterol synthesis and consequently inhibiting corticosterone synthesis [59]. Furthermore, based on the fact that bee pollen has a high carbohydrate content and that comfort food with a similar percentage carbohydrate content is able to lower serum corticosterone concentrations, we can speculate whether this contributed to our results [31,60,61]. On the other hand, the potential anti-stress effect achieved by carbohydrate-rich foods consumption is not supported by the results of another study conducted by Zeeni et al., (2015), in which two types of diets—high carbohydrate and high carbohydrate enriched with highly palatable products—were used in stressed rats. Indeed, rats supplemented with the latter one showed significantly lower serum corticosterone levels than rats eating only a carbohydrate-rich diet [44]. It can be concluded that not only the use of a high-carbohydrate diet but also its additional enrichment was responsible for the reduction of corticosterone concentrations. Since in the current study the intake of whey protein resulted in lower levels of hormone excreted in urine in comparison with bee pollen supplementation, we speculate that whey protein consumption is more effective in affecting adrenal function. Indeed, it has previously been reported that the production of corticosterone in rats is at least partially regulated by diet protein intake [62,63]. Additionally, Makkar et al., (2016) found that supplementation with 0.5% whey protein caused a noticeable decrease in serum corticosterone levels in poultry [64] and similarly, Greco et al., (1982) observed that a high protein diet caused a decrease in serum corticosterone levels in rats [65]. Nonetheless, to understand the potential properties of whey protein on adrenal function, we need to look at its individual bioactive fractions such as lactoferrin and lactalbumin [22]. Maekawa et al., (2017) found that intraperitoneal administration of bovine lactoferrin to rats resulted in a decrease in serum corticosterone levels [66]. Furthermore, another prior experiment showed similar effects to lactoferrin administration [67].

Although our study is, to the best of our knowledge, the first one evaluating the impact of bee pollen and whey protein supplementation on adrenal histology and function, we are aware of its several limitations. First, we regard small sample sizes as a substantial restriction factor. Moreover, urine samples were collected while the rats were in metabolic cages. Although they were held in them for only 24 h, they might have been significantly stressed due to new housing and solitude. Additionally, the implementation of other stress sources in rats will allow us to draw more reliable results. What is equally important, the entire concept of the study topic followed the growing interest of functional foods consumption among people, as well as frequent experience of stress. Hence, in order to verify the effects of these substances on human organisms, future studies should be conducted on this target group.

## 5. Conclusions

The histological structure and functioning of the adrenal glands are a reflection of the impact of stress on the body. Application of exogenous factors including diet enrichment may modify some observed stress-derived changes. We noticed that bee pollen and whey protein have a significant effect on the reduction of urine corticosterone concentrations, which strongly supports their anti-stress value. Additionally, the observed decrease in the percentage of pyknotic nuclei in particular layers in both of the non-running supplemented groups in comparison to the non-running control may suggest the protective and beneficial effects of bee pollen and whey protein consumption on adrenal glands. Overall, the intake of bee pollen and whey protein seems to have limited but promising potential for stress reduction. Nevertheless, as we mentioned above, since the primary objective is to prove the effects of these substances on the human body, there is a need for further experiments.

## Figures and Tables

**Table 1:** The contribution of lipid droplets to the total selected area within the different layers of the cortex.

Group	I	II	III	IV	V	VI	*p* ^A^
Zona glomerulosa	0.03	0.03	0.03	0.03	0.03	0.03	0.7612
Zona fasciculata	0.04	0.02	0.02	0.03	0.03	0.02	0.0516
Zona reticularis	0.03	0.03	0.03	0.03	0.02	0.03	0.1043

^A^**^—^**Kruskal–Wallis test. Note: I—non-running, non-supplemented group (control); II—running, non-supplemented group (control); III—non-running, whey-protein-supplemented group; IV—non-running, bee-pollen-supplemented group; V—running, whey-protein-supplemented group; VI—running, bee-pollen-supplemented group.

**Table 2:** The values of the measurements (median, IQR) by group.

Group	I	II	III	IV	V	VI	*p* ^A^
Nucleus diameter [μm]							
Zona glomerulosa	3.88, 0.905	4.12, 1.25	4.14, 0.843	4.74, 1.15	4.57, 1.16	5.21, 1.22	<0.0001
Zona fasciculata	5.23, 1.03	4.94, 1.50	5.53, 1.12	5.83, 1.38	6.13, 1.31	6.23, 1.11	<0.0001
Zona reticularis	4.54, 0.84	4.70, 1.04	4.44, 0.57	4.20, 0.66	5.00, 0.61	5.22, 0.62	<0.0001
Medulla	5.43, 0.75	6.38, 0.86	6.02, 0.75	6.63, 0.58	6.14, 0.81	6.70, 0.95	<0.0001
Other measurements [μm]							
Sinusoid width	2.97, 0.82	4.24, 1.20	2.98, 1.16	4.08, 1.18	4.36, 2.06	3.72, 1.67	0.0009
Sinusoid epithelium thickness	1.40, 0.37	1.22, 0.29	1.18, 0.28	1.09, 0.30	1.54, 0.38	1.28, 0.32	0.0057
Sinusoid epithelial cell nucleus thickness	2.40, 0.46	2.79, 0.53	2.36, 0.48	2.74, 0.49	2.81, 0.59	2.71, 0.58	0.0025
Capsule thickness	14.03,1.90	11.58,2.80	14.24,2.74	16.01,4.60	15.17, 3.59	14.25, 4.04	0.0019

**^A^^—^**Kruskal–Wallis test. Note: I—non-running, non-supplemented group (control); II—running, non-supplemented group (control); III—non-running, whey-protein-supplemented group; IV—non-running, bee-pollen-supplemented group; V—running, whey-protein-supplemented group; VI—running, bee-pollen-supplemented group.

**Table 3:** Mean optical density of Masson’s trichrome staining by group—measurement of the extent of fibrosis.

Group	I	II	III	IV	V	VI	*p* ^A^
Zona glomerulosa	0.40 ± 0.10	0.37 ± 0.05	0.43±0.08	0.28 ± 0.05	0.34 ± 0.07	0.45 ± 0.13	0.0003
Zona fasciculata	0.45 ± 0.09	0.31 ± 0.03	0.38±0.04	0.30 ± 0.02	0.32 ± 0.04	0.43 ± 0.09	<0.0001
Zona reticularis	0.45 ± 0.05	0.38 ± 0.08	0.38±0.03	0.45 ± 0.07	0.34 ± 0.07	0.52 ± 0.11	<0.0001

**^A^^—^**Kruskal–Wallis test. Note: I—non-running, non-supplemented group (control); II—running, non-supplemented group (control); III—non-running, whey-protein-supplemented group; IV—non-running, bee-pollen-supplemented group; V—running, whey-protein-supplemented group; VI—running, bee-pollen-supplemented group.

**Table 4:** Mean corticosterone values by group.

Group	I	II	III	IV	V	VI	*p* ^A^
Corticosterone in urine [ng/mL]	74.51 ± 16.12	64.23 ± 28.69	23.89 ± 7.80	36.60 ± 19.38	83.51 ± 37.24	88.99 ± 26.83	0.0037
Corticosterone in feces [μg/mg]	0.84 ± 0.33	1.68 ± 0.46	0.91 ± 0.11	1.98 ± 0.74	1.21 ± 0.72	1.24 ± 0.79	0.0612
Total daily corticosterone excretion [μg]	5.74 ± 2.16	8.51 ± 3.14	8.28 ± 5.13	11.10 ± 4.71	7.70 ± 3.40	12.58 ± 1149	0.4016

**^A^^—^**Kruskal–Wallis test. Note: I—non-running, non-supplemented group (control); II—running, non-supplemented group (control); III—non-running, whey-protein-supplemented group; IV—non-running, bee-pollen-supplemented group; V—running, whey-protein-supplemented group; VI—running, bee-pollen-supplemented group.

**Table 5:** The mean percentage of pyknotic nuclei.

Group	I	II	III	IV	V	VI	*p* ^A^
Zona glomerulosa	18.65	8.39	9.64	7.01	10.23	7.00	<0.0001
Zona fasciculata	13.68	8.38	7.26	7.09	6.46	4.09	0.0003
Zona reticularis	14.97	7.40	6.96	7.71	4.01	2.88	0.0001

**^A^^—^**Kruskal–Wallis test. Note: I—non-running, non-supplemented group (control); II—running, non-supplemented group (control); III—non-running, whey-protein-supplemented group; IV—non-running, bee-pollen-supplemented group; V—running, whey-protein-supplemented group; VI—running, bee-pollen-supplemented group.

## Data Availability

Data are contained within the article.
